# Recurrent Non-cirrhotic Hyperammonemic Encephalopathy Due to Complicated Urinary Tract Infection: A Case Report

**DOI:** 10.7759/cureus.39579

**Published:** 2023-05-27

**Authors:** Sagar Pandey, Myo Myint Tun, Shwe Yee Htet, Bhawana Chhetri, Nabin K C

**Affiliations:** 1 Internal Medicine, One Brooklyn Health/ Interfaith Medical Center, Brooklyn, USA; 2 Internal Medicine, Nepal Medical College Teaching Hospital, Kathmandu, NPL; 3 Pulmonary and Critical Care Medicine, One Brooklyn Health/ Interfaith Medical Center, Brooklyn, USA

**Keywords:** proteus mirabilis, urease-producing bacteria, altered mental status, clinical case report, complicated uti, non-cirrhotic hyperammonemia

## Abstract

Hyperammonemic encephalopathy (HE) can be broadly defined as an alteration in the level of consciousness due to elevated blood ammonia level. While hepatic cirrhosis is the most common cause of HE, non-hepatic causes like drugs, infections, and porto-systemic shunts can also lead to the presentation. In this case, we highlight an unusual occurrence of recurrent non-cirrhotic HE from obstructive urinary tract infection (UTI) with urea-splitting micro-organisms in an elderly male patient. The patient exhibited altered mentation, and elevated ammonia levels with normal hepatic function at presentation. Urine culture revealed Proteus mirabilis resistant to extended spectrum beta-lactamases (ESBL). Successful management of obstructive UTI was achieved through Foley’s catheterization and intravenous (IV) antibiotics, resulting in the resolution of HE. This outcome further supports the significance of UTI as a potential cause of hyperammonemia. Thus, UTI as one of the non-hepatic causes of hyperammonemia should always be explored among elderly patients presenting with altered mentation.

## Introduction

Encephalopathy is a clinical condition characterized by a state of altered mental status (AMS) presenting as confusion, disorientation, behavioral changes, or other cognitive impairments with or without inflammation of the brain [1]. Hyperammonemia is the accumulation of ammonia in blood above normal physiologic levels with levels >200 umoL/L correlating with neurotoxic effect [2]. Etiologically, hyperammonemic encephalopathy (HE) can be caused by hepatic failure, inborn errors of metabolism, and other causes like drugs, infections, portosystemic shunts, etc. [3]. Ammonia can penetrate the blood-brain barrier and can cause cerebral edema, astrocyte dysfunction, and synaptic dysregulation leading to mental status change, disorientation, somnolence, confusion, and unconsciousness comprising HE [3,4]. Urease-positive organisms like Proteus mirabilis, Klebsiella pneumoniae, Providencia stuartii, Staphylococcus saprophyticus, etc. generate ammonia via hydrolysis of urea into ammonia and carbamate [5]. Our body has regulatory mechanisms to eliminate elevated ammonia levels under normal physiologic conditions, such as the conversion of ammonia into water-soluble urea in the liver and subsequent excretion in urine, along with detoxification of ammonia into glutamine which occurs in the liver and astrocytes [6]. However, in the case of urinary tract infection (UTI) with urea-splitting organisms, urea excreted in urine is converted back to ammonia and reabsorbed into the systemic circulation, causing hyperammonemia [7]. Here, we present an unusual presentation of obstructive UTI with recurrent non-cirrhotic HE in an elderly patient with a history of bladder outlet obstruction.

## Case presentation

A 69-year-old male patient with a past medical history of adrenocortical insufficiency, atrial fibrillation (AF), benign neoplasm of the pituitary gland, gastroesophageal reflux disease (GERD), hypertension, hypothyroidism, obesity, seizures and history of bladder outlet obstruction (five months ago) on Foley’s catheter was brought to the emergency department from a nursing home with chief complaint of AMS for two to three days. At presentation, the patient was lethargic, minimally communicative with incomprehensible words, disoriented to time, place, and person, and unable to provide pertinent medical history. According to the caretakers, the patient was alert, communicative, able to feed himself, and used to ambulate with the help of a wheelchair before the onset of symptoms. However, further details about the symptoms could not be obtained due to the patient’s mental status. The nursing home caretakers denied any history of trauma/fall, fever, cough, respiratory distress, vomiting, or diarrhea. A review of nursing home medications revealed that the patient was on oral atorvastatin 40 mg once daily, levetiracetam 250 mg twice a day, hydrocortisone 10 mg in the morning and 5 mg in the evening, tamsulosin 0.4 mg at night, finasteride 5 mg once daily, lactulose 45 ml three times a day as needed, baclofen 10 mg twice a day, simethicone 125 mg oral once daily, levothyroxine 25 mcg oral once daily and vitamin tablets. Furthermore, the patient had a Foley’s catheter placed for bladder outlet obstruction secondary to bulbourethral stricture on previous admission. The catheter was taken out at the patient's request one month before the current presentation at the nursing home. As the patient was wearing diapers, information about the patient’s ability to pass urine could not be reliably obtained. 

The patient had a similar presentation five months ago with AMS with elevated serum ammonia levels. CT abdomen and pelvis done at that time showed a distended bladder with bilateral hydroureteronephrosis raising suspicion of bladder outlet obstruction. After repeated failed attempts of per urethral Foley's catheterization, cystoscopy-guided Foley catheter placement over guidewire was done, draining foul-smelling turbid urine. A urethrotomy was not attempted at that time due to the risk of bleeding as the patient was on Eliquis for atrial fibrillation. The patient was discharged with a Foley catheter in situ.

At the time of presentation, the patient's vital signs were stable. On examination, the patient was muttering incomprehensible words, disoriented to time, place, and person with a Glasgow coma scale (GCS) of 13 (eye opening: four, best verbal response: four, and best motor response: five) and able to maintain his airway. The patient was able to maintain an oxygen saturation of 96% on room air. Bilateral pupils were equal and reactive, and cardiovascular and respiratory examinations were within normal limits. Abdominal examination revealed a mildly distended, soft non-tender abdomen with normal bowel sounds. Suprapubic tenderness was not elicited on examination. However, the examination was conducted following drainage of 800 ml of urine via per urethral straight catheterization. On neurological examination, the patient had bilateral normal and symmetrical reflexes. However, tone, power, sensation, and cranial nerve examination could not be done as the patient was in an altered mental state and was not able to follow verbal commands. Unfortunately, due to the patient's altered mentation and resistive behavior, fundoscopy could not be done at the time of presentation.

Laboratory parameters at presentation are depicted in Table 1. 

**Table 1:** Laboratory parameters at presentation AST: aspartate transaminase, ALT: alanine transaminase, ALP: alkaline phosphatase, PT: prothrombin time, INR: international normalized ratio

Lab parameters	At admission	Normal range
Hemoglobin	14.5	13-17 gm/dL
Hematocrit	44	39-53%
White blood cells	5.6	4.5-11 10x6/uL
Blood urea nitrogen	42	7-25 mg/dL
Creatinine	1.2	0.7-1.3 mg/dL
Sodium	148	136-145 mEq/L
Potassium	3.4	3.5-5.1 mEq/L
Serum ammonia	271	16-53 umol/L
AST	41	13-39 U/L
ALT	21	7-52 U/L
ALP	75	34-104 U/L
Total protein	9.1	6.4-8.9 g/dL
Albumin	4.3	3.5-5.7 g/dL
Total bilirubin	0.7	0.3-1.0 mg/dL
PT	12	9.8-13.4 secs​​​​​​
INR	1.01	0.85-1.15
Lactic acid	0.7	0.5-1.9 mmol/L
High sensitivity troponin	6.6	0.0-35.0 ng/L

**Figure 1:**
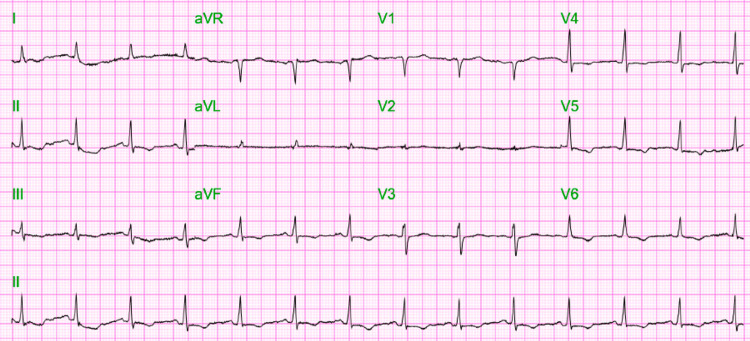
Electrocardiogram (EKG) at presentation showing normal sinus rhythm

Electrocardiogram (EKG) showed normal sinus rhythm with a heart rate of 80 beats per minute, PR interval of 168 ms, QRS duration of 90 ms, and QTc of 452 ms (Figure 1). 

**Figure 2:**
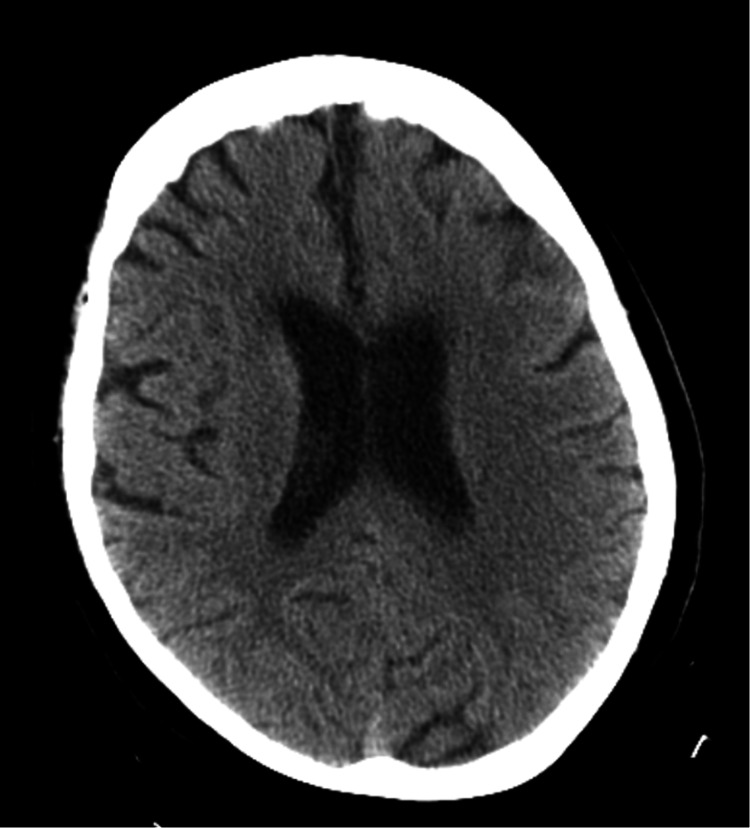
Non-contrast computed tomography of the head at admission negative for acute intracranial bleed, infarct, or cerebral edema

**Figure 3:**
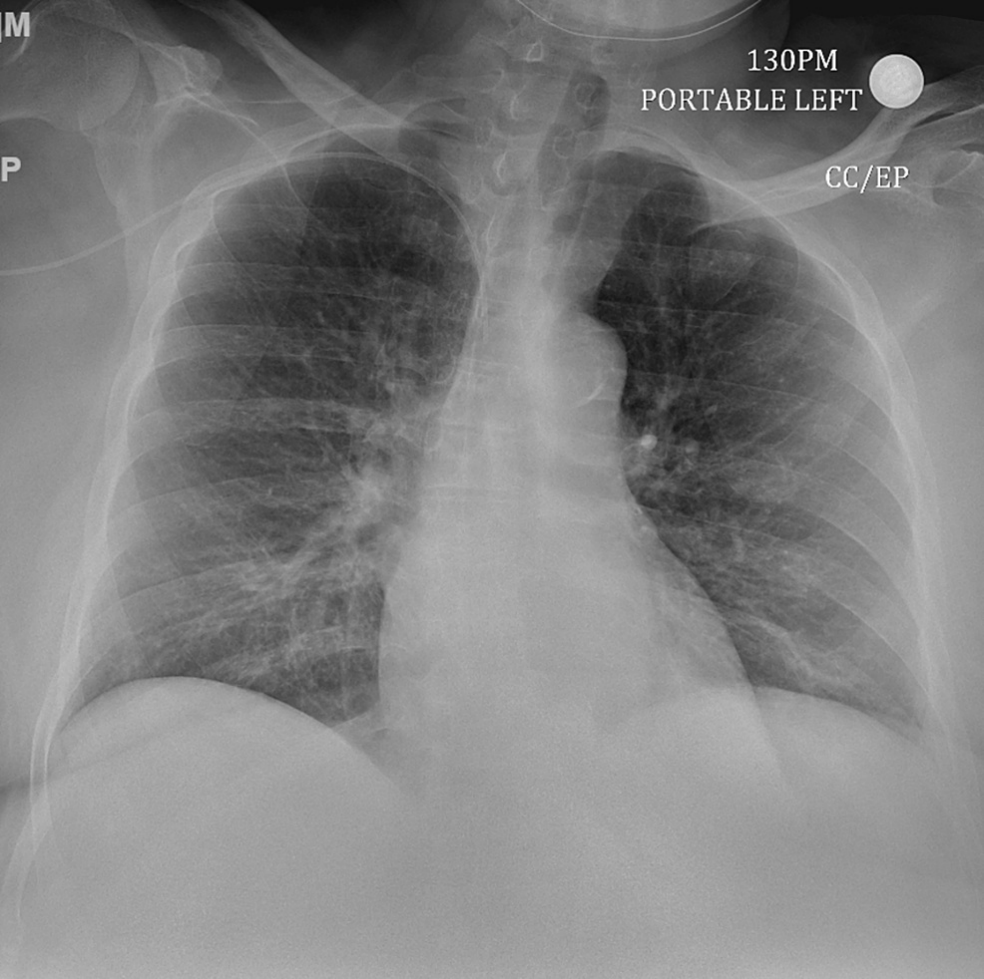
Chest X-ray at admission negative for cardiopulmonary abnormality

Non-contrast computed tomography (NCCT) head showed no evidence of acute intracranial bleed, infarct, or any evidence of cerebral edema and the chest X-ray was negative for any radiographic evidence of cardiopulmonary abnormality (Figures 2-3).

**Figure 4:**
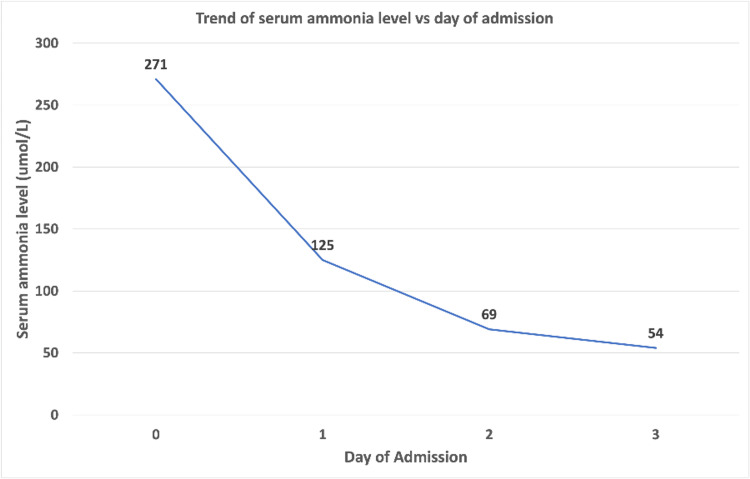
Trend of serum ammonia level vs day of admission

The patient was kept nil per oral (NPO) with intravenous (IV) fluids and started on lactulose, rifaximin along with IV meropenem and vancomycin. Under urology guidance, a Foley's catheter was placed with difficulty which drained clear urine. Urology was consulted and the patient was planned for elective optical urethrotomy after medical stabilization on an outpatient basis. IV vancomycin was discontinued on Day 2, and the patient was continued on IV meropenem as urine culture was growing extended-spectrum beta-lactamase (ESBL) Proteus mirabilis sensitive to meropenem. The ammonia level trended down on Day 3 (Figure 4) and the patient’s mentation also improved. Lactulose and rifaximin were discontinued after the ultrasound abdomen showed no evidence of liver cirrhosis. The patient was discharged on Day 6 of admission with a peripherally inserted central line (PICC) line to complete a total two-week course of IV meropenem for a complicated UTI.

## Discussion

Here we discuss an atypical presentation of obstructive UTI with recurrent HE. Although hepatic cirrhosis is the most common cause of hyperammonemia, a normal liver enzyme level, negative history of significant alcohol intake (with negative serum alcohol level), negative hepatitis panel, and normal liver ultrasound made a hepatic etiology unlikely in our patient. The urine toxicology screen was negative. Even though hypernatremia during presentation could be argued as a cause for altered mentation, acute hypernatremia with mild elevation of sodium level (sodium: 148 mEq/L) made it a less plausible explanation to justify the presentation. Hemato-oncological disorders like multiple myeloma and chemotherapy were ruled out as causes for hyperammonemia with complete blood counts within normal range and absence of pertinent medical history. Negative history of use of medications like valproic acid, glycine, carbamazepine, and ribavirin excluded drug-induced hyperammonemia. Furthermore, hyperammonemia in an elderly patient with a negative past medical history of similar presentation at a young age made inborn errors of metabolism an unlikely cause. In addition, the normalization of ammonia levels following treatment of UTI with IV antibiotics along with the placement of Foley's catheter and isolation of Proteus mirabilis on urine culture further corroborated the association. 

Proteus mirabilis accounts for 3% of hospital-acquired infections and 44% of catheter-associated UTIs (CAUTI) in the USA [8,9]. Several agents and host factors have been attributed to the development of CAUTI. One of the most important host factors include the presence of an indwelling catheter which can lead to the introduction of pathogens into the urinary tract while placing the catheter, can impair the natural flushing action of the urinary stream due to continuous bladder drainage via the catheter and provide a reservoir for bacterial replication due to incomplete drainage [10]. Proteus mirabilis-associated virulence factors include the presence of flagella for movement from the point of entry to the bladder and kidneys, fimbriae and adhesins for biofilm formation, the presence of urease to increase urine pH favorable for its growth and leading to precipitation of urinary stones (struvite and apatite), bacterial toxins like hemolysins leading to tissue damage, etc [10]. The resulting buildup of ammonia leads to osmotic swelling of astrocytes due to an increase in intracellular glutamine levels as well as increasing the reactive oxygen species and disrupting the synaptic neurotransmitter regulation [6,11]. While the liver can convert ammonia into water-soluble urea to excrete in the urine, the presence of urease-positive organisms in urine made the conversion futile. Urea excreted in urine would be converted back into ammonia by the Proteus in the urine and reabsorbed into the bloodstream. Furthermore, urinary obstruction amplified the mechanism even further by allowing more time for Proteus to split urea into ammonia and in addition, provide more time for systemic reabsorption of ammonia [7]. Lastly, the long-standing obstruction with a distended bladder wall and increased surface area provided enough time for the diffusion of ammonia into the perivesical circulation which bypasses the portal circulation, thereby leading to persistently elevated serum ammonia levels [12]. Immediate resolution of hyperammonemia following the placement of Foley’s catheter further supports this theory. 

## Conclusions

Hyperammonemia secondary to UTI is one of the conditions that can be rapidly treatable and manageable if diagnosed in an appropriate and timely manner. Therefore, clinicians should always watch out for non-cirrhotic causes of HE like UTI in patients presenting with altered mentation with or without the concomitant presence of liver disease. This is especially relevant in the case of elderly patients with multiple comorbidities where altered mental status could be easily attributed to a worsening of baseline dementia or underlying chronic medical condition.
